# Physicochemical and Inflammatory Analysis of Unconjugated and Conjugated Bone-Binding Carbon Dots

**DOI:** 10.1021/acsomega.3c07653

**Published:** 2023-12-15

**Authors:** Quan Chau, Lesly Corado-Santiago, Shannon Jones, Jonathan Dattelbaum, Isaac Skromne

**Affiliations:** † Department of Biology, University of Richmond, 138 UR Drive, Richmond, Virginia 23173, United States; § Department of Chemistry, University of Richmond, 138 UR Drive, Richmond, Virginia 23173, United States

## Abstract

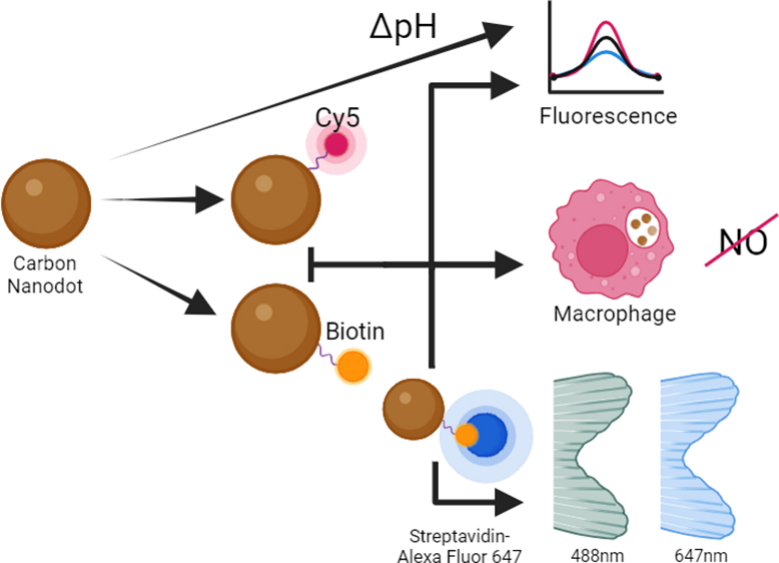

Carbon nanodots (CDs) have drawn significant attention for their potential uses in diagnostic and therapeutic applications due to their small size, tissue biocompatibility, stable photoluminescence, and modifiable surface groups. However, the effect of cargo molecules on CD photoluminescence and their ability to interact with tissues are not fully understood. Our previous work has shown that CDs produced from the acidic oxidation of carbon nanopowder can bind to mineralized bone with high affinity and specificity in a zebrafish animal model system. Using this model, we investigated the impact of loading Cy5 and biotin cargo on CDs’ photoluminescence and bone-binding properties. We report that CD cargo loading alters CD photoluminescence in a pH- and cargo-dependent manner without interfering with the CDs’ bone binding properties. In a reciprocal analysis, we show that cargo loading of CDs does not affect the cargo’s fluorescence. Significantly, CDs do not trigger nitric oxide production in a mouse macrophage assay, suggesting that they are noninflammatory. Together, these results further support the development of carbon nanopowder-derived CDs for the precise delivery of therapeutic agents to bone tissue.

## Introduction

Carbon dots (CDs) are a large and diverse group of photoluminescent nanoparticles defined by their small size (∼10 nm) and carbonized core. Over the past decade, CDs have drawn significant attention for diagnostic and therapeutic use because, in contrast to metal-based quantum nanodots, CDs are compatible with biological tissues.^1−4^ Furthermore, CDs have modifiable core structures and surface groups that offer unique pharmacological opportunities and challenges. The CDs’ chemical, structural, and mechanical properties can be tuned by changing the origin material and preparation method to better suit the intended biological applications.^3,4^ In one synthesis method (“bottom-up”), polymerization of small carbon-containing molecules generates particles with crystalline cores and outer hydroxyl and carboxyl groups^5−8^ that, in the presence of nitrogen-containing molecules, can also display surface amine groups.^7,9,10^ In an alternative synthesis method (“top-down”), large carbonaceous materials are stripped down to a nanoparticle size using lasers, electricity, or acidic oxidation treatments to generate amorphous cores surrounded by hydroxy and carboxylic groups.^5,11−13^ Both methods produce a highly photoluminescent CD that can interact with tissues, inhibit bacterial growth, or deliver antitumor drugs to different extents.^1,2,7−11^ While the efficacy of the interactions can be further adjusted by adding metal and nonmetal elements to the CDs during synthesis,^7−14^ frequently, the outcomes are difficult to predict because each CD formulation possesses distinct physicochemical properties that make them unique.^1,2^ Thus, the exceptional properties that make CD uniquely suited to tackle modern diagnostic and therapeutic challenges also demand the individual investigation of their potential.^1,2^

CDs synthesized from carbon nanopowder under acidic oxidation treatment have shown high affinity and specificity for zebrafish bone tissue,^6,15,17^ garnering interest for their potential use as a drug delivery method to treat bone-related diseases such as osteoporosis.^17^ Zebrafish bones are physiologically similar to mammals and can heal or regenerate quickly,^18,19^ allowing rapid assessment of CD’s interaction with bone. These studies have revealed that CD binding to bones occurs in the zone of appositional growth,^17^ depends on the bone mineralization state,^15^ does not interfere with homeostatic or regenerative growth,^17^ and is nontoxic for larvae or adults.^6,17^ While the precise mechanism of bone binding is still unknown, two lines of evidence suggest that this binding is likely dependent on the abundance of surface hydroxyl and carboxylic groups. In experiments where the number of carboxylic groups was reduced, CDs did not bind to bones,^6^ whereas modifications that increased these groups also enhanced the CDs ability to bind to bones.^20,21^ More importantly, we do not know how cargo-loading affects CD bone binding affinity and drug efficiency and how CDs interact with the immune system. Addressing the effect of cargo on CD’s physicochemical and biological properties is essential for further developing CDs as theragnostic agents.

Toward developing CDs as agents to deliver drugs to bones, we characterized the physicochemical properties of unconjugated and conjugated CDs and tested their pro-inflammatory potential. Here, we report that cargo loading and changes in pH impact the photoluminescent properties of CDs, sometimes enhancing and sometimes diminishing their fluorescence emission. This finding has important implications for the use of CDs as diagnostic tools. Significantly, cargo loading does not interfere with CD deposition on bones. Finally, we show that cell-cultured macrophages can internalize CDs and that this event does not trigger an immune response. Together, these results further support the potential theranostic use of carbon nanopowder-derived CDs for treating bone disease.

## Results and Discussion

### Synthesis and Characterization of CD-Cy5 and CD-Biotin

**Figure 1:**
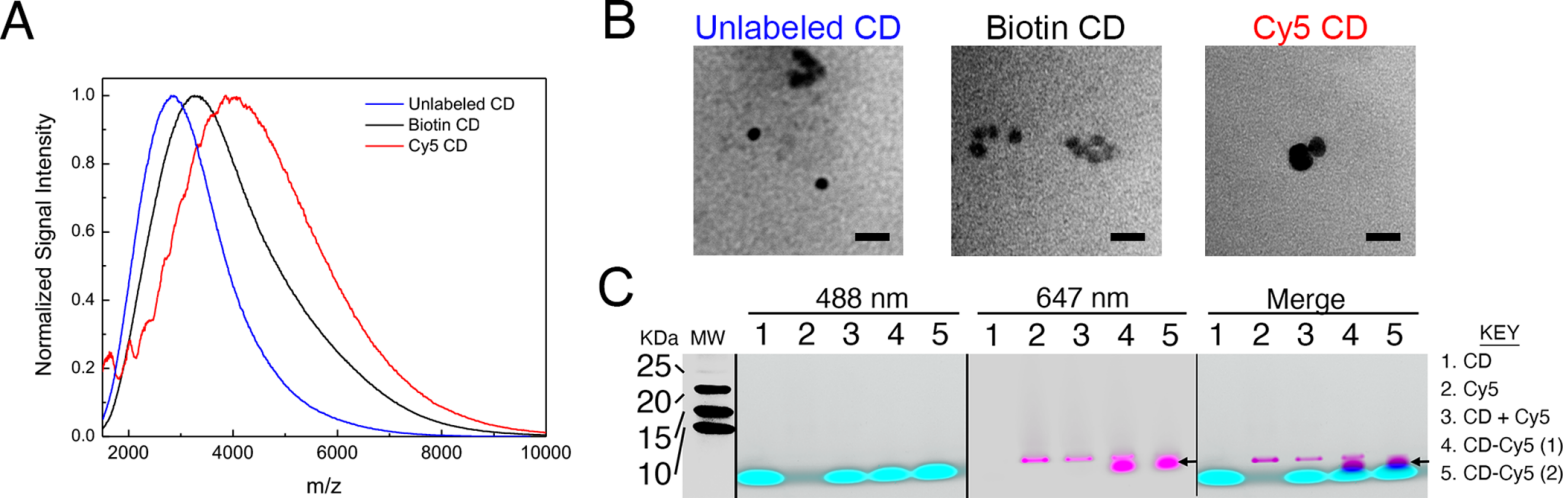
Characterization of biotin- and Cy5-loaded CDs. (A) MALDI mass spectrometry of CD (∼3k Da; blue), CD-Biotin (∼3.5k Da; black), and CD-Cy5 (∼4.2 kDa; red). (B) TEM images of unlabeled, biotin-loaded, and Cy5-loaded CDs. Scale bars are 20 nm. (C) SDS-PAGE chromatograph of CD, Cy5, CD mixed with Cy5, and two different preparations of CD-Cy5 (1 and 2) imaged under 488 nm (CD, pseudocolored cyan) and 647 nm (Cy5, pseudocolored magenta) excitation. CD-Cy5(1) and CD-Cy5(2) were purified using different conventional chromatography methods (1, size exclusion; 2, anion exchange). The far-right gel is a composite of merged images. The arrow indicates new molecular species that appear after EDC/NHS conjugation. The molecular weight (MW) ladder is in kDa.

To test CDs’ ability to deliver cargo molecules to bone tissues, we cross-linked the CDs to Cy5 or biotin using classic EDC/NHS chemistry, purifying cargo-loaded CDs from unreacted material using conventional chromatography methods. We selected Cy5 because its robust infrared fluorescence has minimum overlap with the CDs’ intrinsic green and red fluorescence^15^ and biotin because of its broad biotechnological uses and available detection tools (e.g., antibodies, streptavidin). Using MALDI, TEM, and DLS methods, we confirmed that the CDs had a molecular weight of ∼3 kDa and a size of ∼10 nm (Figure 1A,B and not shown), similar to our previously published preparations.^16^ Employing the same methods, we determined the extent of Cy5 and biotin loading to CDs. Loading CDs with biotin and Cy5 increased the MW of CDs by ∼0.5 and ∼1.2 kDa, respectively (Figure 1A), without altering their particle size (Figure 1B and data not shown). Given the molecular weight of biotin (0.244 kDa) and Cy5 (0.716 kDa), and the size distribution of cargo-loaded CDs (Figure 1A), we calculated that each CD carried between 1 and 3 cargo molecules. While the number of cargo molecules per CD may appear low, it was sufficient for monitoring cargo delivery to bones (see below).

To further characterize the CD conjugates, we next used SDS-PAGE. Using this method, we observed that all of the conjugates ran faster than the 10 kDa standard (Figure 1C), in agreement with our mass spectrometry analysis. In the gel, under white light, CDs were detected as brown bands (not shown) that fluoresced green under 488 nm light (Figure 1C, pseudocolored cyan). Cy5, on the other hand, ran as a sharp band that was only visible under 647 nm light (Figure 1C, pseudocolored magenta). CD-Cy5 conjugates prepared in the presence of EDC/NHS showed a new product that was not present when EDC/NHS was omitted from the reaction. This new product showed fluorescent upon excitation with 488 and 647 nm light (Figure 1C arrows), indicating CD-Cy5 conjugation. We observed this unique, double fluorescent product in samples purified using conventional size exclusion (CD-Cy5(1)) and anion exchange (CD-Cy5(2)) chromatography, with the latter being more effective than the former in removing unincorporated Cy5. These results suggest that EDC/NHS cross-links 1–3 cargo molecules to one CD particle under these experimental conditions. For CD-Cy5, this number of cargo molecules is sufficient for fluorescent detection.

### CD Fluorescence Is Dependent on pH and Cargo

**Figure 2:**
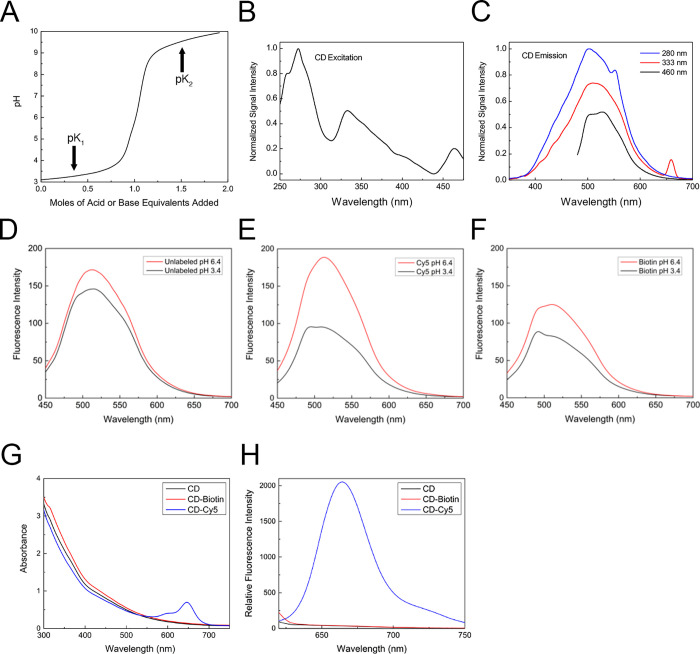
pH and cargo influence CD fluorescence. (A) Titration curve of a 0.05 mg/mL CD suspension using HCl and NaOH. The two p*K*_a_s found were p*K*_1_ = 3.4 and p*K*_2_ = 9.6. (B) CD excitation signal intensity was determined across different wavelengths. (C) CD emission spectra were recorded at 280, 333, and 460 nm excitation wavelengths at pH 6.4. (D-F) CD (D), CD-Cy5 (E), and CD-biotin (F) fluorescence spectra were obtained under 420 nm excitation light at pH values of 3.4 (black line) and 6.4 (red line). Absorbance (G) and emission (H) spectra of CD, CD-biotin, and CD-Cy5 show Cy5 absorption and emission peaks in the 600–700 nm range.

CDs have abundant carboxylic acid groups on the surface that aid in their aqueous dispersion and cargo loading.^6^ To determine the number and behavior of the carboxylic groups, we titrated 50 mL of a 0.05 mg/mL aqueous suspension of CD with HCl or NaOH. At the onset of the experiment, the suspension had a pH of ∼5.2. Upon titration, we identified two p*K*_a_’s, p*K*_1_ at pH 3.4 and p*K*_2_ at pH 9.6 (Figure 2A). It is possible, however, that the second p*K*_a_ may be the result of excess NaOH and not due to additional CD deprotonation events. We also determined the equivalence point at pH 6.4, calculating 3.4 μmol of carboxylic acid per milligram of CD.

The fluorescent properties of molecules can be significantly affected by their synthesis method, pH, and side groups.^22−24^ When dispersed in water (pH ∼ 5.2), our preparation of CDs strongly absorbed light in the 250–460 nm range and demonstrated excitation–dependent emission with a maximum wavelength of around 510 nm (Figure 2B,C). This excitation and emission spectra are similar to previous preparations.^15^ To test the effect of pH on CD fluorescence, we adjusted the pH of the suspensions to 3.4 (p*K*_1_) and 6.4 (equivalence point) and measured the CD fluorescence spectra (Figure 2D–F). Fluorescence emission spectra were collected by using excitation at 420 nm to avoid any overlap of the emission peak with the water Raman peak. Lowering the pH from 6.4 to 3.4 resulted in a slight reduction in the emission intensity without changing the overall shape of the emission curve, suggesting that the fluorescence of CD tolerates acidic pH.

We also analyzed the fluorescent properties of conjugated CD-Cy5 and CD-biotin at two different pHs. At pH 6.4, the addition of Cy5 or biotin to CDs caused opposite changes in CD fluorescence, with Cy5 increasing and biotin decreasing excitation-independent emission (Figure 2D–F). At pH 3.4, however, both modifications reduced the CD fluorescence similarly. These results suggest that adding cargo molecules to CDs can alter the CDs’ fluorescent properties in a cargo-dependent manner. Thus, for each CD-cargo pair, the direction and magnitude of the change need to be empirically determined. Furthermore, these results also suggest that cargo loading exacerbates the dampening effect of a low pH on CD fluorescence.

We next examined whether CD could alter the properties of a cargo molecule. To this end, we analyzed the absorption and emission spectra of Cy5 in unconjugated and Cy5-conjugated CD at pH 6.4. Similar to Cy5, CD-Cy5 showed an excitation peak at 651 nm and an emission peak at 670 nm (Figure 2G,H). These peaks were not observed in CD and CD-biotin (Figure 2G,H). These results suggest that Cy5 fluorescence properties are not affected by its conjugation to CD. Further work will be necessary to determine whether this phenomenon is restricted to Cy5 or is generalizable to other fluorescent molecules.

### Cargo-Loaded CDs Retain Bone-Binding Properties

**Figure 3:**
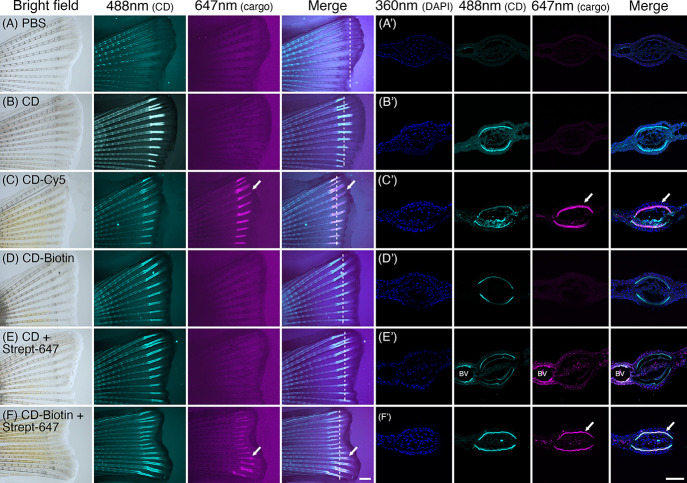
Conjugated CDs deliver cargo to bones. Caudal fins of zebrafish injected 4 days after amputation with (A) PBS, (B) CD, (C) CD-Cy5, (D) CD-Biotin, (E) CD and Streptavidin-Alexa 647, and (F) CD-Biotin and Streptavidin-Alexa 647 and imaging the following day. Deposition of CDs and cargo (Cy5 or Streptaviding-Alexa-647) was observed at 488 and 647 nm, respectively. Anterior is to the right and dorsal to the top. Arrows indicate areas of cargo deposition, and dashed lines are the planes of histological sectioning. Scale bar is 500 μm. (A’–F’) Cryosection images were observed under 360 nm (DAPI, nuclei), 488 nm (CD), and 647 nm (cargo) excitation light. Arrows indicate the areas of cargo deposition. BV is a blood vessel that shows blood autofluorescence. Scale bar is 50 μm.

Our previous work using a zebrafish caudal fin regeneration assay has shown that unconjugated CDs bind with high affinity to bone and not to other tissues.^17^ To test if cargo-loaded CDs retain bone-binding properties after conjugation, we repeated this experiment using CD-Cy5 and CD-Biotin, monitoring bone binding using CD’s intrinsic fluorescence. As previously reported,^17^ injection of unconjugated CDs into adult fish that have been regenerating their caudal fin rays for 4 days results in strong fluorescence in areas of bone growth when compared to PBS-injected controls (Figure 3A,B). We observed similar strong bone fluorescence after injecting CD-Cy5 and CD-biotin (Figure 3C–F). Examination of CD-Cy5 injected fish upon illumination at 648 nm revealed colocalization of Cy5 infrared fluorescence with the CD signal (Figure 3D), indicating that CDs can deliver the Cy5 cargo to bones. To detect biotin delivery to bones, we injected CD-biotin mixed with streptavidin-Alexa633 followed by an analysis of the distribution of the fluorescent labels. Since the binding of streptavidin (52 kDa protein) to biotin is one of the strongest noncovalent interactions known in biology,^25^ the presence of fluorescently labeled streptavidin in bones would indicate the presence of biotin cargo attached to CDs. Compared to fish injected with CD-biotin only (Figure 3E), injection of the CD-biotin and streptavidin-Alexa633 mix resulted in strong fluorescence of the bones at both 488 and 648 nm wavelengths (Figure 3F). Significantly, we observed similar bone regeneration dynamics across control and experimental conditions in agreement with our previous observations that CDs are inert and nontoxic.^17^ These results show that CDs can deliver biotin cargo to bones and proteins of up to 52 kDa in size. Furthermore, these results also show that adaptor molecules like biotin can bypass the need to conjugate cargo molecules to the CDs’ surface directly.

### CDs Do Not Activate Macrophage Inflammatory Response

**Figure 4:**
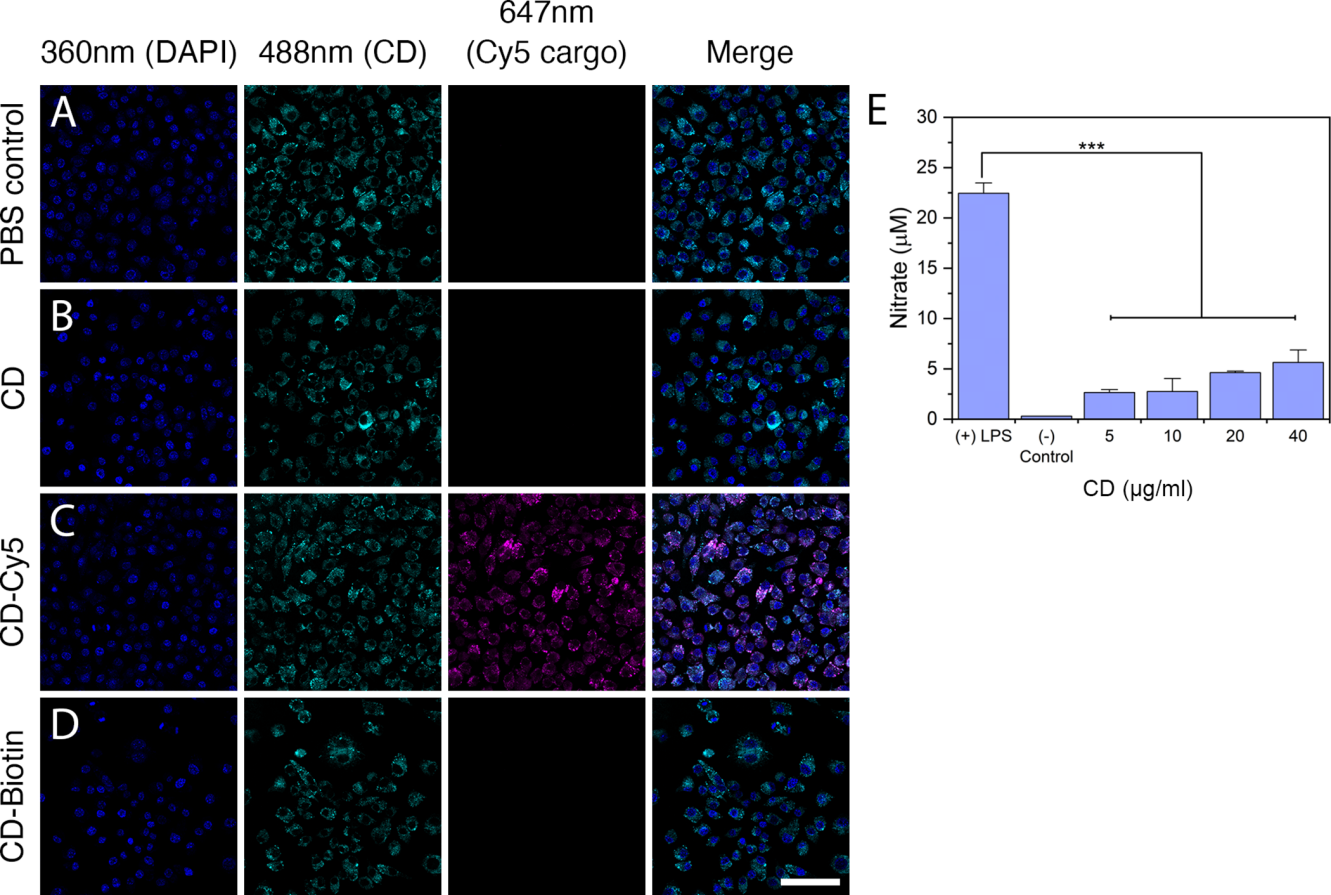
Macrophages internalize CDs without activating inflammatory programs. Mouse RAW 264.7 macrophages internalize CDs. Macrophages were exposed for 6 h to (A) PBS, (B) CD, (C) CD-Cy5, and (D) CD-Biotin, costained with DAPI, and imaged at 360, 488, and 647 nm to respectively identify nuclei (blue), CDs (cyan), and Cy5 (cargo; magenta). While autofluorescence in the 488 nm channel precludes direct analysis of CD internalization, examination in the 647 nm channel revealed the internalization of CD-Cy5. Scale bar is 50 μm. (E) Nitric oxide (NO) production by mouse RAW 264.7 macrophages exposed to different CD concentrations. NO production was measured as released nitrate (μM). Lipopolysaccharide (LPS; immune response activator) was used as a positive control, and PBS as a negative control. Data represent the average ± SEM of three independent experiments and were analyzed for statistical significance using a one-way ANOVA followed by Tukey’s posthoc test (***p < 0.001).

The potential use of CDs as therapeutic agents led us to consider their interaction with the immune system. We tested CDs’ ability to induce an immune response using a standard *in vitro* assay that relies on nitric oxide production by murine RAW 264.7 macrophages when exposed to immunogens. We first determined whether macrophages could take up unconjugated and conjugated CDs following 6 h exposure. We observed that macrophages exposed to control, CD, CD-biotin, and CD-Cy5 conditions had similar cytology (Figure 4A–D), suggesting that CDs do not have cytotoxic effects. To determine the CD distribution inside the cells, we resorted to monitoring the Cy5 fluorescence in CD-Cy5 as macrophages displayed low levels of fluorescence at 488 nm that precluded the direct analysis of CD distribution (Figure 4A). We found that CD-Cy5 distributed in puncta reminiscent of vesicles underneath the cell’s surface membrane (Figure 4C), suggesting that macrophages can internalize CDs without causing discernible morphological changes.

We next examined whether macrophage exposure to CDs resulted in the activation of pro-inflammatory pathways and the release of nitric oxide (measured as μM of released nitrate).^26,27^ Macrophages produced high amounts of nitric oxide when exposed to bacterial lipopolysaccharides (LPS; positive control; Figure 4E).^26,27^ Exposure to CDs, however, induced significantly low levels of nitric oxide relative to controls (2 independent experiments, *n* = 3 per treatment, *p* < 0.001; Figure 4E). The highest concentration tested was 40 μg/mL, as this is the highest concentration of CD we can safely deliver to zebrafish.^17^ Together, these results suggest that CDs have minimal or no pro-inflammatory activity.

In conclusion, the data presented here further support and expand the potential use of CDs in diagnostic and therapeutic use in bone tissue. Our previous regeneration studies found that CDs are nontoxic and do not interfere with bone growth and remodeling processes.^17^ Here, we report that CDs have no or low pro-inflammatory activity in a macrophage assay (Figure 4), an advantageous characteristic for any molecule being developed as a diagnostic or therapeutic tool. Furthermore, CDs remain highly fluorescent near physiological pH even when coupled to cargo (Figure 2). Thus, CDs can simultaneously be used as vehicles to bring therapeutic agents to bones and diagnostic agents to monitor the proper delivery of the agents to the target tissue. Significantly, CDs can effectively deliver covalently or noncovalently attached cargo molecules up to 18 times their size, if not bigger (e.g., 3 kDa CDs vs 54 kDa streptavidin-Alexa633; Figure 3). However, it is essential to note that the molecular nature of the cargo can significantly impact CD’s fluorescence (Figure 2D–F). This effect has important implications for bioimaging applications, as the signal of the CDs may change based on the loaded cargo molecule. Altogether, our data further support CDs as a versatile, nontoxic, noninflammatory drug carrier and monitor system with the potential to treat adult skeletal diseases and bone-related injuries.

## Experimental Procedures

### Carbon Dot Synthesis and Conjugation

Carbon nanodots (CDs) were synthesized from carbon nanopowder (MilliporeSigma; #633100) under acid reflux (1:3 ratio of 15.9 M nitric acid to 18 M sulfuric acid) and purified using previously reported procedures.^16,17^ After neutralization, the CD solution was dried in a SpeedVac concentrator at 35 °C to obtain CD in powder form. The physicochemical properties of the CDs were verified after each preparation, as previously described.^16^

CDs were conjugated to either Cy5 or biotin. To conjugate CDs, 2.5 μL of 13 mM Cy5-amine (in DMSO; Lumiprobe; no. 1333C0) or 5 μL of 14.4 mM Amine-PEG3-Biotin (in H_2_O; ThermoScientific; #21347) was mixed with 1.11 mg/mL CD in phosphate buffer (0.2 M Na_3_PO_4_; pH 8). Then, 4 mg of EDC (AnaSpec. Inc.; #AS-29855) was added to the mix with stirring at room temperature overnight in the dark. The resulting solution was purified by following one of two conventional chromatography methods. Both methods were equally effective in separating CD-conjugates from the unconjugated material. One method utilized size exclusion chromatography using Affi-Gel 10GD prepacked columns (Biorad 10DG Desalting Column; #7322010) and water as the eluant. The second method utilized anionic exchange chromatography using 2 mL of a DEAE Sepharose matrix column (Cytiva; #17071901) equilibrated with phosphate buffer at pH 6.3. CDs were eluted using NaCl solutions of increasing concentrations. Fractions containing CDs were desalted and concentrated using centrifugal filter units (Millipore, #UFC800324). After purification, CDs were dried using a Speedvac, weighed, and resuspended in water at the appropriate concentration prior to use.

### Molecular Weight and Size Characterization

We characterized the molecular weight of the CD by MALDI-MS and SDS-PAGE. For MALDI-MS, the different CD preparations were suspended in a matrix of sinapinic acid before analysis (Shimadzu Biotech, AXIMA Confidence). For SDS-PAGE, all CD preparations were resuspended in water at 2 mg/mL, and 18.75 μL was mixed with 6.25 μL of 80% glycerol and separated using 4–20% SDS-PAGE (BioRad; #4561093). We compared the band positions relative to those of a protein ladder standard to determine the molecular weight of the CD preparations. To identify the CD-biotin and CD-Cy5 conjugates, gel images were analyzed at 488 nm (CD) and 647 nm (Cy5) using a gel documentation system (BioRad, Chemidoc MP Imaging System). To determine the size of the CD, we used dynamic light scattering (DLS) and transmission electron microscopy (TEM). DLS was performed using a DynaPro NanoStar II instrument from Wyatt Technology. For TEM, 7 μL of a 2 mg/mL CD suspension was dried onto a Formvar-coated copper grid. CD images were then collected by using a JEOL JEM-1400flash microscope.

### Titration and Spectrophotometric Analysis

We determined the number of carboxyl groups on the CD surface by titrating 50 mL of a CD suspension (0.05 mg/mL in water) with 0.1 M HCl or NaOH in 20 μL increments. We titrated CDs from two different synthesis reactions in triplicate using a SympHony SB70P pH meter (VWR). We characterized the spectrophotometric properties of 2 mg/mL CD solutions at different pH values using an FP-6200 spectrofluorometer (Jasco).

### Cell Culture Assays

Murine RAW 264.7 macrophages (ATCC) were grown following standard cell culture practices, as previously described.^26^ The day before the experiment, 3.3 × 10^6^ cells/well were plated in a 6-well plate. The following day, cells were exposed to 0.2 μg/mL LPS (positive control; Sigma-Aldrich; *E. coli* 055:B5) or different concentrations of CDs in fresh media (negative control, 0 μg/mL CD). After 24 h, 50 μL of supernatant was analyzed for nitric oxide (NO) production measured as released nitrate (μM) using Greiss reagent as previously described.^26^ To determine whether cells uptake CDs, murine RAW 264.7 macrophages were incubated and grown on a coverslip overnight and then incubated for 6 h with different CD conjugates at 20 μg/mL before imaging.

### Zebrafish Care, Tail Amputation, Injection, and Bone Staining

Zebrafish of the transparent line Casper (*mpv*^*17a9*^*; mitfa*^*w2*^)^28^ were obtained from the Zebrafish International Resource Center (Eugene, OR) and maintained at the University of Richmond animal facility following standard husbandry protocols.^29^ The Institutional Animal Care and Use Committee reviewed and approved all of the protocols and procedures. Caudal fin regeneration assays and injection protocols were carried out as previously described.^17^ Briefly, fish 4–6 months of age were anesthetized in 0.2 mg/mL Tricaine (pH 7.0) until unresponsive to touch and placed on an inverted glass Petri dish covered with Parafilm, and a sterile blade was used to cut 40 mm away from the distal tip of the caudal fin. For injection, fish were anesthetized and weighed to standardize the mass of CD injected per body weight (μg/g). Fish were then positioned on a wet sponge under the microscope and injected intraorbitally using a 36G Nanofill microsyringe attached to an electronically controlled micropump (WPI,UMP3 UltraMicroPump).^30^ After manipulations, the fish were allowed to recover for 30 min and returned to the animal facility, where they received standard care for the duration of the experiment.

### Histology, Microscopy, and Image Processing

CD incorporation into caudal fin bones was analyzed in whole and sectioned tissue. Four days postinjection, zebrafish were anesthetized, placed on a glass Petri dish, and imaged using a dissecting Stereo V.20 and compound AxioExaminer Z.1 microscopes (Zeiss). For histology, the regenerated portion of the caudal fins was removed and fixed in 4% paraformaldehyde for at least one h before rinsing them in PBS and placed in 30% sucrose solution overnight at 4 °C. Fins were placed in Tissue Plus OCT Compound (Fisher HealthCare; #4585) and frozen at −25 °C. Sections were taken at 5 μm using a cryostat (ThermoFisher, HM525 NX) and placed on charged glass slides (VWR; #16004–406) coated with Tissue Capture Pen (TedPella, 22310). Slides were air-dried at 4 °C for at least 24 h before being rehydrated with PBS, placed in mounting media with DAPI (Vector Laboratories, H-1200), and imaged on an Olympus IX83 inverted confocal microscope. We used the same microscope to image the fixed macrophages. All images were processed using the open-source program ImageJ from the NIH. Figures were assembled using Adobe Photoshop.

## Notes

All animal protocols and procedures were reviewed and approved by the University of Richmond Animal Care and Use Committee (protocol #20–10–001).
